# Unregulated medication use and complications: a case study of prolonged self-treated tuberculosis in Nepal

**DOI:** 10.1186/s12879-023-08637-7

**Published:** 2023-10-05

**Authors:** Eliz Achhami, Seshkanta Lamichhane, Satyam Mahaju, Ashim Kandel, Anubhav Poudel, Rabina KC

**Affiliations:** 1 Sukraraj Tropical & Infectious Disease Hospital, Kathmandu, Nepal; 2 Bir Hospital, Kathmandu, Nepal

**Keywords:** Tuberculosis, Self-treatment, Visual impairment, Nepal

## Abstract

**Background:**

Tuberculosis (TB) is a global public health issue, particularly in resource-constrained countries like Nepal. This case report highlights the consequences of prolonged self-treatment and non-compliance with TB management protocols, emphasizing the need for increased awareness and intervention.

**Case Presentation:**

A 50-year-old male from Nepal self-medicated with anti-tubercular drugs for 13 years after completing the recommended course of treatment. He experienced worsening symptoms, including respiratory distress and visual impairment. Upon evaluation, he was diagnosed with chronic cavitary pulmonary aspergillosis. The patient received comprehensive treatment, including antifungal therapy, steroids, antibiotics, and respiratory support, resulting in significant improvement.

**Conclusions:**

This case highlights the dangers of self-treatment and non-compliance with TB management protocols. It emphasizes the importance of patient education, awareness programs, and regular follow-up to ensure treatment adherence and detect complications. The case also reveals gaps in the DOTS (Directly Observed Treatment, Short Course) program, including the need for improved surveillance, and a multidisciplinary approach. The ease of over-the-counter purchase of anti-tubercular drugs in Nepal contributed to the patient’s prolonged self-medication, highlighting a concerning. The complications arising from prolonged self-medication underscore the need for increased awareness, intervention, and patient education in TB management. Improving patient education, raising awareness about the risks of self-medication, and integrating ophthalmologic evaluations into standard management are essential for better TB control in Nepal.

## Background

Tuberculosis is a multisystem infectious disease that is common throughout the world, particularly prevalent in countries with resource-constrained economies. According to the World Health Organization (WHO) approximately 10.6 million people were diagnosed with TB in 2021 [1]. It is caused by a type of acid-fast and alcohol-fast mycobacteria [2]. As in other developing nations, Nepal faces a significant public health burden in the form of tuberculosis. According to the World Bank, the incidence of tuberculosis in Nepal in 2021 will be 229 per 100,000 people. However, the detection rate of tuberculosis in Nepal is only about 41% [3]. Effective treatment of tuberculosis requires timely diagnosis and adherence to the prescribed treatment regimen. Failure to comply with medical advice or engage in self-treatment can result in treatment failure, the emergence of drug-resistant strains, and complications associated with medication. We present a unique case of prolonged self-treated TB for a long time in Nepal, highlighting the consequences of unregulated medication use, the need for increased awareness and intervention, and the complication of long-term use of antitubercular drugs.

## Case Presentation

A 50-year-old male from the Terai Belt of Nepal presented to the Emergency Department with a complex medical history. The patient was initially diagnosed with sputum positive pulmonary tuberculosis 15 years ago and successfully completed the recommended course of anti-tubercular therapy (ATT) as per the national guidelines. However, two years after completing the treatment, the patient began experiencing symptoms of cough, sputum production, and occasional hemoptysis. Instead of seeking medical advice, the patient chose to self-medicate by purchasing and consuming four tablets of ATT daily from a local pharmacy shop. Each tablet contained 150 mg of Rifampicin, 75 mg of Isoniazid, 400 mg of Pyrazinamide, and 275 mg of Ethambutol. This pattern of self-medication continued for approximately 3–4 months at a time, repeated over the course of the past 13 years.

Six years prior to his current presentation, the patient noticed a gradual decline in his vision. Over time, his vision deteriorated to the point where he could only perceive light. In addition to his visual impairment, the patient had experienced a recent worsening of symptoms, including an increase in cough, sputum production, shortness of breath, and wheezing over the past month. Concerned about his symptoms, he decided to visit Bir Hospital for further evaluation. The patient denied a history of jaundice but reported multiple episodes of nausea, vomiting, and anorexia. He did not complain of weakness, tingling sensation, paresthesia, or joint pain in his limbs.

**Fig. 1:**
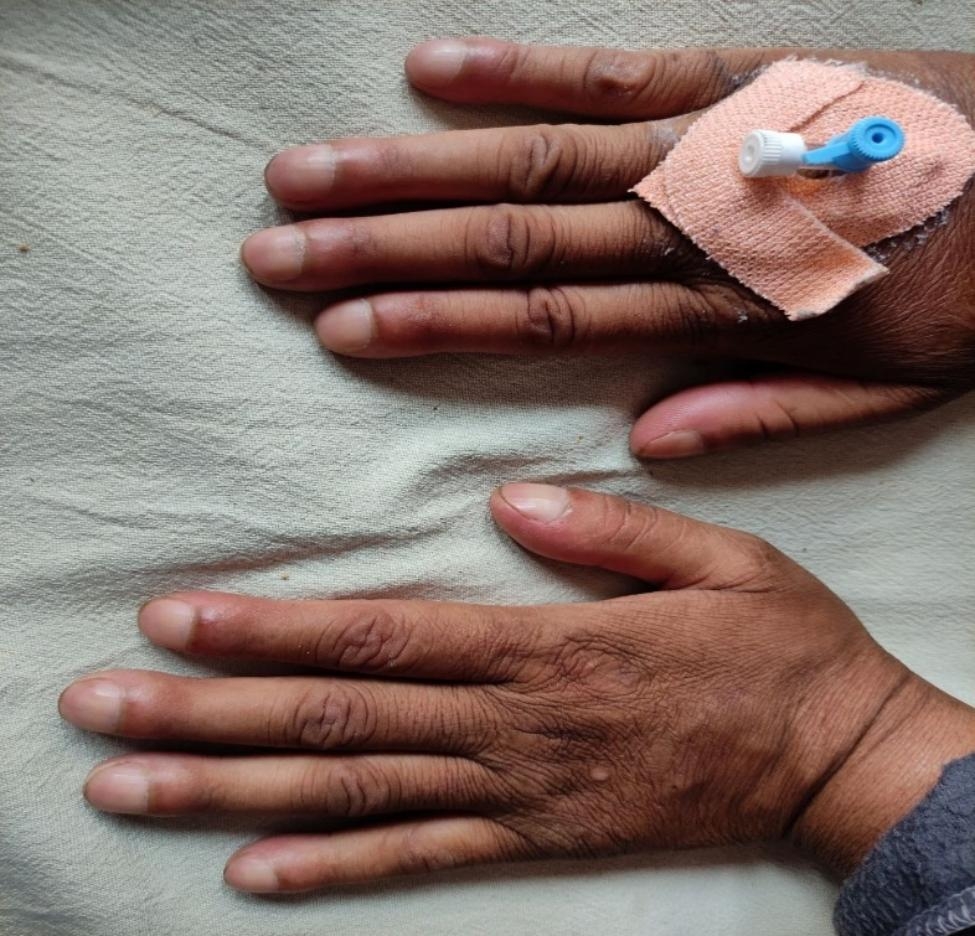
Digital clubbing seen on both hands

Upon examination in the Emergency Department, the patient presented with tachypnea, distress, confusion, and the following vital signs: a blood pressure of 114/86 mmHg, a pulse rate of 96 beats per minute, a respiratory rate of 28 breaths per minute, and an oxygen saturation of 44% in room air. Physical examination revealed that the patient appeared pale, and digital clubbing was observed in all the patient’s digits (Fig. 1). During chest auscultation, decreased breath sounds were detected on the left side of the chest, along with areas of crepitation and diffuse wheezing bilaterally.

Further investigations were conducted to assess the patient’s respiratory status. Arterial blood gas analysis showed a pH of 7.30, a PaCO2 of 65 mmHg, a PaO2 of 75 mmHg with FiO2 of 60%, and an HCO3 of 32. Based on these findings, he was promptly initiated on intravenous antibiotics and steroids to address underlying infections. Additionally, the patient was placed on bi-level positive airway pressure (BiPAP) support. The patient’s laboratory findings at the time of admission and at the time of discharge are summarized in Table 1.

**Table 1:** His blood investigation at time of admission and at the time of discharge

Lab value	Day of Admission	At Discharge	Referance Lab Value
TLC (Total Lymphicyte count)	14,600	9300	4000–11,000
Platelets	300,000	274,000	150,000–400,000
Hemoglobin	10.6 gm/dl	9.4 gm/dl	13–18
RBS (Random blood glucose)	132 mg/dl	116 mg/dl	70–140
Urea	74 mg/dl	24 mg/dl	10–50
Creatinine	1.1 mg/dl	0.8 mg/dl	0.4–1.4
Total Bilirubin	0.7 mg/dl	0.5 mg/dl	0.4-1
Direct Bilirubin	0.2 mg/dl	0.2 mg/dl	0-0.4
ALT (Alanine transaminase)	505 IU/L	184 IU/L	5–35
AST (Aspartate aminotransferase)	523 IU/L	72 IU/L	5–50
ALP (Alkaline phosphatase)	92 IU/L	55 IU/L	35–180
Total Protein	7 gm/dl	6.8 gm/dl	4.5-8
Albumin	2.8 gm/dl	2.6 gm/dl	3.5–5.5
PT (Prothrombin Time )	20 s	17 s	14
INR international normalized ratio)	1.38	1.2	0.8-1.2
Total IgE		> 2500ku/L	Upto 100
Allergen- Aspergillus Fumigatus Specific IgE		3.11	< 0.1
Allergen- Aspergillus Fumigatus Specific IgG		32.3	< 27.0

**Fig. 2:**
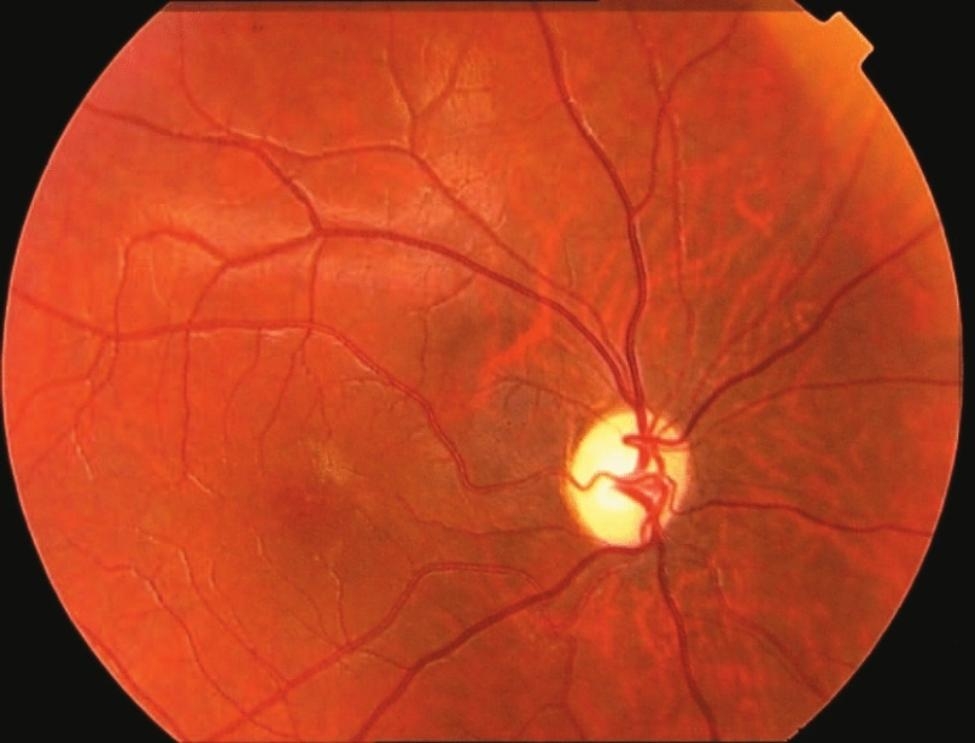
Fundoscopy showing optic disc atrophy

**Fig. 3:**
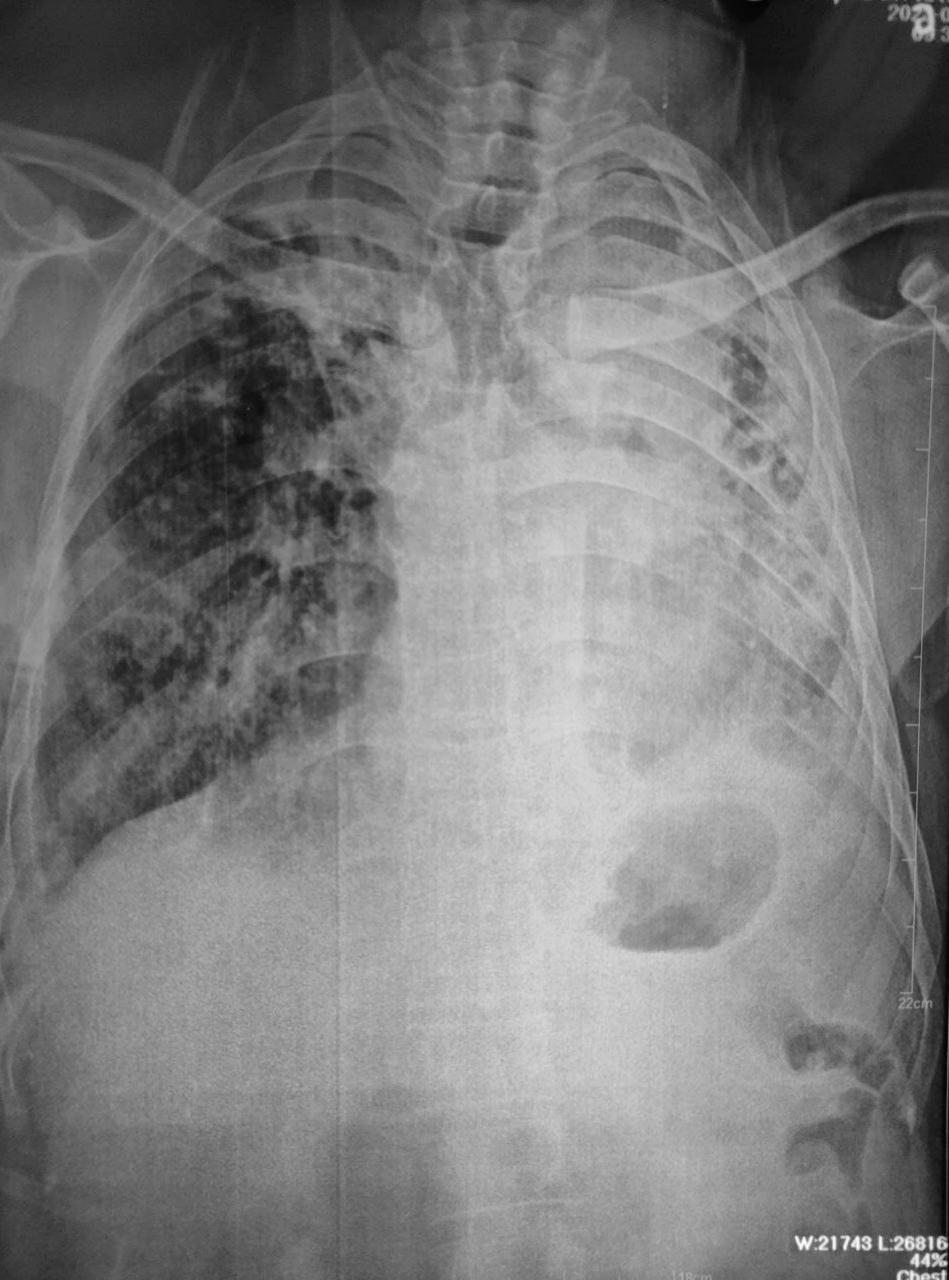
CXR showing presence of bronchiectasis and a few cavities in the lung

**Fig. 4:**
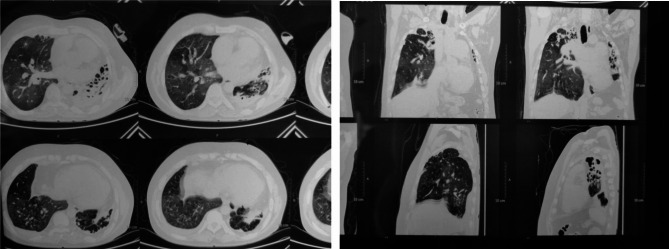
HRCT of chest showing patchy ground glass opacities, cystic bronchiectatic changes indicating post infective sequelae

**Fig. 5:**
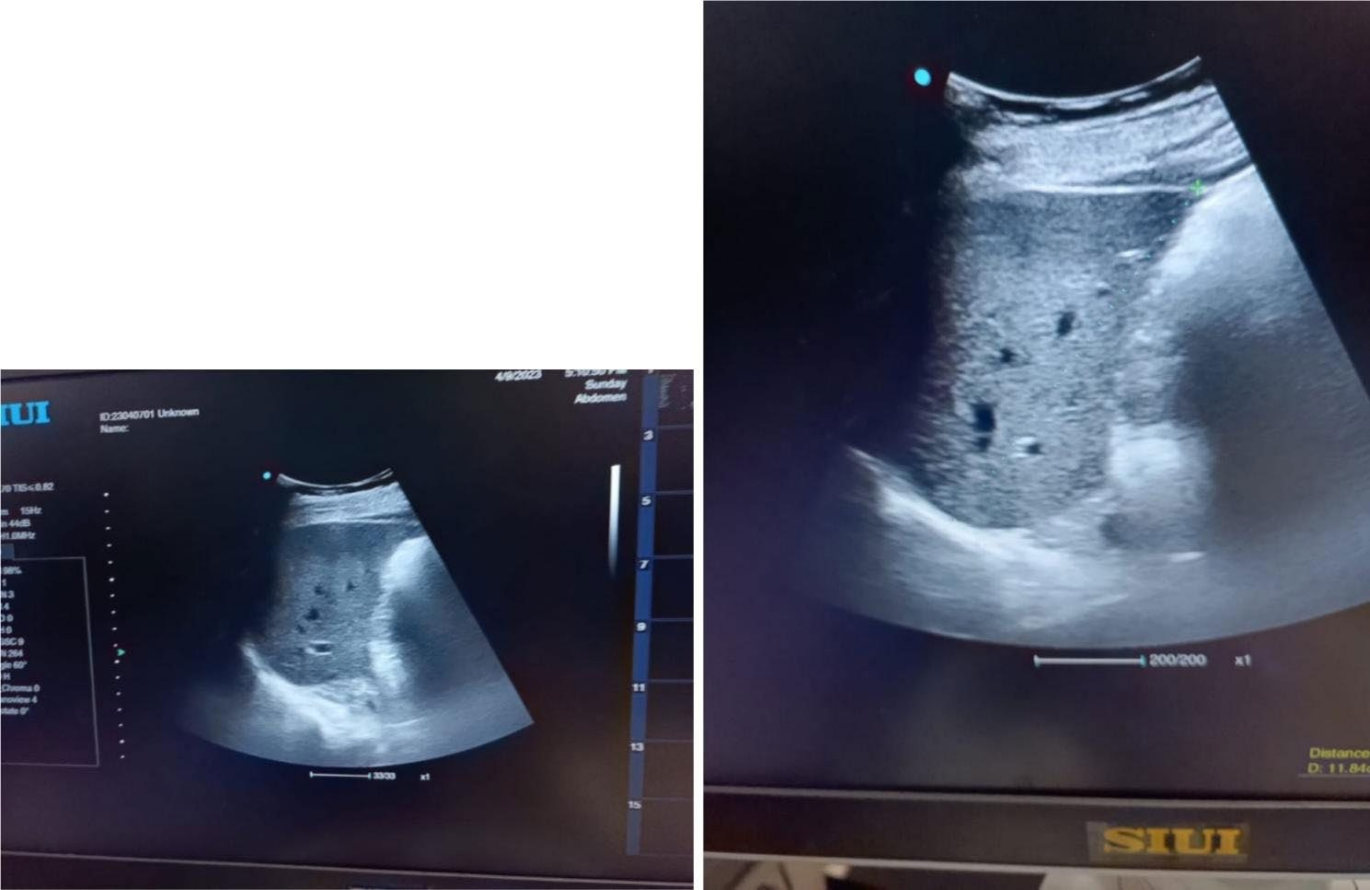
USG showing normal Liver

The patient’s visual impairment was thoroughly assessed through an ophthalmologic evaluation. The Fundoscopic examination of the both eyes revealed bilateral optic disc atrophy (Fig. 2). The patient’s visual acuity was limited to perceiving only light. To investigate the respiratory symptoms and assess the extent of pulmonary involvement, a chest X-ray and a High-Resolution Computed Tomography (HRCT) scan of the chest were performed. The X-ray image (Fig. 3) showed evidence of post-tuberculosis sequelae, which included the presence of bronchiectasis and a few cavities in the lung tissue. The HRCT image (Fig. 4) revealed further evidence of post-tuberculosis sequelae, confirming the presence of bronchiectasis and providing a clearer visualization of the cavities in the lung tissue. These findings indicated the long-term effects of previous tuberculosis infection. Following the radiological investigations, blood analysis was conducted to identify the underlying cause of the pulmonary findings. The blood tests confirmed the presence of chronic cavitary pulmonary aspergillosis. In addition to the radiological investigations of the chest, an ultrasound examination of the abdomen was performed to assess the patient’s liver. Figure 5 shows a normal appearance of the liver with no evidence of focal lesions, hepatomegaly, or other abnormalities.

Following the diagnosis of chronic cavitary pulmonary aspergillosis, he was managed with additional antifungal treatment (itraconazole) along with steroids, antibiotics, and respiratory support. As a result of the comprehensive treatment approach, the patient experienced significant improvement in his condition. Symptoms such as shortness of breath and cough were alleviated, and there was an improvement in oxygen levels and ventilation. Following the successful management of his respiratory distress and the control of the fungal infection, the patient was discharged with a treatment plan that included inhalation bronchodilators, continued use of inhaled steroids, oral antifungal medication, and a tapering dose of oral steroids.

## Discussion and conclusions

This case report emphasizes the dangers of self-treatment and non-compliance with TB management protocols. The patient’s self-medication approach, driven by the resolution of hemoptysis after each medication phase, highlights the lack of awareness regarding the importance of sustained therapy and appropriate drug selection. Prolonged self-medication can lead to treatment failure, and the emergence of drug resistance, resulting in severe systemic complications as well as the dissemination of TB infection within the community. Our case report not only sheds light on the adverse outcomes of prolonged self-treated tuberculosis but also underscores the broader issues that contribute to such situations. One of these issues is the ease of over-the-counter drug purchase, which can lead to inadequate understanding of treatment protocols and potential adverse effects.

Tuberculosis (TB) remains one of the major public health problems in Nepal. According to the annual data of the National Tuberculosis Program (NTP) of Nepal, a total of 32,043 tuberculosis cases were reported and registered with the National Tuberculosis Program (NTP) in 2018/19 (4). The Reported and estimated incidence of tuberculosis cases in Nepal has stagnated for more than decades (CNR 152/100,000 in 2018), but the program is doing its best to detect and treat more tuberculosis cases. Tuberculosis cases were reported from all parts of the country, with the highest number of cases reported in the plains/terais, followed by the hills and mountains. Nepal’s tuberculosis program also fails to detect nearly 28% of its estimated annual number of infected cases, which plays a major role in controlling tuberculosis, of which 20–25% are tied up in the private sector and go unreported [4]. Timely and appropriate treatment is the foundation for the efforts to curb the incidences and prevalence of tuberculosis. Hence, to avoid poor or incomplete treatment, which can lead to relapse, increased transmission, and drug-resistant TB, WHO and the International Union Against Tuberculosis and Lung Disease advocate the use of the Directly Observed Treatment, Short Course DOTS strategy [5].

Since being implemented in early 1996, the Directly Observed Treatment, Short Course (DOTS) strategy for tuberculosis control in Nepal has been provided by all Government Community Health Facilities, including Heath Posts totaling approximately 4,955 [4]. Diagnostic services are provided through identified Microscopic centers (nearly 600 in number). The government of Nepal provides free tuberculosis treatment. DOTS consists of five elements: political commitment; microscopy services; drug supplies; surveillance and monitoring systems and use of highly efficacious regimens; and direct observation of treatment [4]. An integral part of this strategy is direct observation of treatment (DOT)which entails healthcare professionals directly observing patients while they receive anti-tuberculosis therapy for at least the initial two months of treatment [4]. Despite all the challenges, Nepal has recorded treatment success rates of over 90% for DS-TB (drug sensitivity - tuberculosis) and over 70% for DR-TB (drug resistance - tuberculosis). Ongoing efforts include the development of a comprehensive multispectral approach, patient-centered care, active involvement of the private sector, and community-based TB care [4].

The level of tuberculosis (TB) patient care provided by healthcare practitioners is generally substandard, resulting in delayed recovery, an increase in persistent carriers, drug resistance, and a higher TB incidence rate [6]. In addition to surveillance, there is a clear need for patient education and awareness. Many patients in Nepal, especially those from economically disadvantaged backgrounds, have inadequate knowledge regarding TB and its treatment. This lack of understanding leads to lower adherence to treatment and adoption of self-medication practices, as evidenced in this case report. Therefore, it is imperative to implement appropriate patient education and awareness programs are essential to ensure that patients understand the importance of continued treatment, the potential risks of self-medication, and the need for regular follow-up.

This case presented here highlights the critical role of pharmacists in ensuring the proper use of medications and highlights several drawbacks of the DOTS program in Nepal. Firstly, it raises concerns about patient education and awareness. The patient, despite completing the initial course of ATT, chose to self-medicate with inappropriate use of ATT obtained from a local pharmacy shop. This highlights a lack of understanding about the importance of seeking medical advice and following recommended treatment protocols. This self-administration of medications without proper knowledge or medical guidance can lead to incorrect dosing, potentially compromising treatment effectiveness and increasing the risk of adverse effects. Improving patient education and raising awareness about the risks of self-medication are crucial aspects that need attention in the DOTS program. In this case, the patient’s prolonged self-medication could have introduced bias by influencing his perception of symptoms and affecting his reporting of medical history. To mitigate this bias, our medical team meticulously collected information from the patient, corroborated it with diagnostic tests, and considered alternative explanations for his symptoms.

Secondly, the case reveals a gap in surveillance and follow-up mechanisms. The patient continued self-medicating for an extended period of 13 years without proper medical supervision and incomplete treatment adherence. Regular monitoring and follow-up of TB patients are essential to ensure treatment adherence, detect treatment failure or relapse, and provide appropriate interventions. The lack of effective surveillance, and follow up for complications of pulmonary tuberculosis, in this case allowed the patient’s condition to deteriorate significantly, leading to severe respiratory distress and visual impairment.

Additionally, the case emphasizes the importance of a multidisciplinary approach in managing complex TB cases. The patient presented not only with respiratory symptoms but also with visual impairment, suggesting possible ocular involvement. Integrating ophthalmologic evaluations into the standard management of TB patients could aid in the early detection and management of ocular complications, as seen in this case with bilateral optic disc atrophy.

Antituberculous drugs, like many other drugs, cause unwanted side effects, especially when taken at high doses, usually for more than two months [7, 8]. Unfortunately, while these drugs are effective in treating tuberculosis, they cause many side effects, such as visual impairment [9]. For example, ethambutol is known to cause optic neuropathy and optic chiasmosis [7, 10]. Moreover, alarmingly, ethambutol is toxic to retinal ganglion cells via an excitotoxic pathway [11]. In addition, isoniazid is associated with optic neuropathy, peripheral neuropathy, and hepatotoxicity [8, 9]. Rifampicin is known for microsomal enzyme induction and hepatotoxicity, whereas pyrazinamide is associated with hepatotoxicity, gastrointestinal disturbances, and hyperuricemia [9]. Side effects of concomitant medications for tuberculosis can be related to drug dose and duration of use or can be idiosyncratic [9]. Socioeconomic problems and stigmas are associated with this disease. Understanding treatment plans and being compliant with the services provided is also problematic [12]. It also reflects one of the key elements of care: the provider-patient relationship. Patients find it difficult to seek TB treatment because they do not trust the TB program and most of them do not have sufficient knowledge about TB itself [13]. Toxic optic neuropathy is caused by toxin damage to the optic nerve. Nutritional deficiencies, particularly B vitamins, and folic acid, can cause or exacerbate toxic optic neuropathy. There is no evidence of racial or gender bias in toxic or diet-related ocular neuropathy [14].

In conclusion, our case report not only underscores the consequences of prolonged self-treatment but also highlights the underlying challenges in tuberculosis management, particularly within the context of self-medication and over-the-counter drug acquisition. The patient’s decision to self-medicate with inappropriate use of anti-tubercular drugs resulted in treatment failure, severe systemic complications. It highlights the urgent need for increased awareness, intervention, and patient education in TB management. The lack of awareness and misunderstanding of the disease can lead to incorrect dosing, compromising treatment effectiveness, and increasing the risk of adverse effects. Improving patient education and raising awareness about the risks of self-medication are crucial aspects that need immediate attention in the Directly Observed Treatment, Short Course (DOTS) program. Strengthening regulatory measures for pharmacies, including stringent checks and controls, can help prevent the over-the-counter dispensing of potent medications such as anti-tubercular drugs. Additionally, the case highlights the need for a multidisciplinary approach in managing complex TB cases. Integrating ophthalmologic evaluations into the standard management of TB patients could aid in the early detection and management of ocular complications. By addressing these aspects, we can strive towards better outcomes and more effective control of tuberculosis.

## Data Availability

All the data generated or analyzed during this study are included in the manuscript.

## Abbreviations

TB: Tuberculosis
ATT: anti-tubercular therapy
DOTS: Directly Observed Treatment, Short Course
BiPAP: bi-level positive airway pressure
HRCT: High-Resolution Computed Tomography
NTP: National Tuberculosis Program
